# Exploration of Solid Solutions and the Strengthening of Aluminum Substrates by Alloying Atoms: Machine Learning Accelerated Density Functional Theory Calculations

**DOI:** 10.3390/ma16206757

**Published:** 2023-10-19

**Authors:** Jingtao Huang, Jingteng Xue, Mingwei Li, Yuan Cheng, Zhonghong Lai, Jin Hu, Fei Zhou, Nan Qu, Yong Liu, Jingchuan Zhu

**Affiliations:** 1 School of Materials Science and Engineering, Harbin Institute of Technology, Harbin 150001, China; 2 National Key Laboratory for Precision Hot Processing of Metals, Harbin Institute of Technology, Harbin 150001, China; 3 National Key Laboratory of Science and Technology on Advanced Composites in Special Environments, Harbin Institute of Technology, Harbin 150001, China; 4 Center for Analysis, Measurement and Computing, Harbin Institute of Technology, Harbin 150001, China; 5 State Key Laboratory for Environment-Friendly Energy Materials, School of Materials Science and Engineering, Southwest University of Science and Technology, Mianyang 621010, China

**Keywords:** aluminum substrate, single atoms, mechanical properties, explainable machine learning, density function theory

## Abstract

In this paper, we studied the effects of a series of alloying atoms on the stability and micromechanical properties of aluminum alloy using a machine learning accelerated first-principles approach. In our preliminary work, high-throughput first-principles calculations were explored and the solution energy and theoretical stress of atomically doped aluminum substrates were extracted as basic data. By comparing five different algorithms, we found that the Catboost model had the lowest RMSE (0.24) and lowest MAPE (6.34), and this was used as the final prediction model to predict the solid solution strengthening of the aluminum matrix by the elements. Calculations show that alloying atoms such as K, Na, Y and Tl are difficult to dissolve in the aluminum matrix, whereas alloy atoms like Sc, Cu, B, Zr, Ni, Ti, Nb, V, Cr, Mn, Mo, and W exerted a strengthening influence. Theoretical studies on solid solutions and the strengthening effect of various alloy atoms in an aluminum matrix can offer theoretical guidance for the subsequent selection of suitable alloy elements. The theoretical investigation of alloy atoms in an aluminum matrix unveils the fundamental aspects of the solution strengthening effect, contributing significantly to the expedited development of new aluminum alloys.

## 1. Introduction

Aluminum and aluminum alloy materials have been studied by a wide range of scholars because of their advantages, such as being lightweight and having good plasticity. Furthermore, the electrical conductivity of aluminum is surpassed only by copper, silver, and gold. As a result, aluminum is extensively utilized in various industries, including aerospace, automotive, and food packaging. The material exhibits favorable processing characteristics and can be machined and fabricated using numerous forming methods. Being non-magnetic, it has been particularly valuable in the production of electronic and electrical devices. Moreover, aluminum alloys are widely employed in heat sinks and high-temperature equipment due to their superior thermal conductivity. Aluminum alloys are extensively used in the aerospace and automotive sectors for the manufacturing of structural components and housings, among other applications. Consequently, aluminum alloys have garnered significant experimental [1,2] and computational attention [3], highlighting their potential for a wide range of applications.

In the industry, aluminum is commonly referred to as “pure aluminum” when it has a purity greater than 99.00 wt.%. However, its strength is rather low, being only about 50 MPa. The mechanical properties of aluminum alloys show a significant improvement with the addition of Cu, Si, and Mn elements [4]. Research has shown that the inclusion of Cu can modify the microstructure of Al-Mg-Si alloys during the solid solution process and alter the alloy aging precipitation sequence. Nevertheless, excessive amounts of Cu can lead to grain boundary polarization, reducing intergranular corrosion resistance [5]. Trace additions of Sn and In can effectively suppress the natural aging of aluminum alloys, and Cu, Ge, and Zn alloying into the β-phase can improve the stability of the alloy. Furthermore, Xiao et al. [6] performed computational analyses on the precipitated phases during the aging of aluminum alloys and found that Mg and Zn atoms could become polarized at grain boundaries, altering both the bonding environment and binding energy at the interface. Mn shows some degree of solid solution in the alloy [7], but mainly exists in the form of the Al6Mn phase; the Al6Mn phase can act as a nucleation site for the β-phase, facilitating uniform nucleation within the crystal and therefore enhancing the alloy’s corrosion resistance. Likewise, the strengthening alloying element Zr is commonly used to improve the microstructure of aluminum alloys [8,9]. Some researchers have also investigated the simultaneous addition of Fe and Cu to enhance the mechanical properties of Al-Si cast alloys [10]. In recent years, rare earth elements have gained more attention from scholars due to their strategic advantages in the context of a new technological revolution.

Numerous experimental advancements have been made in the investigation of alloying elements as dopants for aluminum. However, aluminum alloys lack a systematic theoretical framework to explain the mechanism behind the action of these elements. Recently, density functional theory has emerged as a widely adopted tool in material design due to its ability to expedite the design process, improve calculation accuracy, and enhance result reliability. Although first-principles calculations are highly accurate, they are computationally intensive, thereby inhibiting progress in new material development. Therefore, this paper employs machine learning techniques [11,12] to accelerate first-principles calculations [13,14] and conduct a comprehensive investigation into the micromechanical behavior of aluminum substrates doped with alloyed atoms. Five distinct machine learning algorithms were utilized to establish mathematical models based on a dataset generated from density functional theory calculations. The models were subsequently compared in terms of decision factors and root mean square errors, allowing for the selection of the most suitable model. Finally, the machine learning models were employed to predict the solution energy and micromechanical behavior of the aluminum matrix doped with other atoms. The amalgamation of first-principles calculations with machine learning algorithms yielded highly accurate forecasts of the solution energy and its impact on the micromechanical behavior of individual atoms in the aluminum matrix. This paper is organized into three sections: Section 2—Computational details, Section 3—Results and Discussion, and Section 4—Conclusions.

## 2. Computational Details

### 2.1. Crystal Structure and Calculation Method

As an FCC structure, the Al(111) surface is known for having the highest density and lowest surface energy, making it a commonly used surface for constructing computational models in the literature [15,16]. In this paper, the aluminum system doped with alloy consists of 72 atoms, with 71 aluminum atoms and 1 alloy atom, as shown in Figure S5. The stretched model includes a 20 Å vacuum layer. To determine the interfacial fracture strength and weakest path, we adopted a method of interface fracturing, with 11 sampling points spaced at a strict interval of 0.5 Å each. Computational simulation techniques based on first-principles have been extensively applied to investigate metal–alloy interfaces [17,18,19]. In our calculations, we utilized the Cambridge Sequential Total Energy Package (CASTEP) [20] simulation package to perform first-principles electronic structure calculations employing density functional theory (DFT) with the generalized gradient approximation (GGA) [21,22,23]. Specifically, we employed the Perdew–Burke–Ernzerhof (PBE) function [24,25], which is a functionally parametrized GGA function. A plane-wave basis set with a 470 eV cutoff energy was used [3]. The integrable Brillouin zone (BZ) was sampled using a 5 × 5 × 5 Monkhorst Pack center k-point grid, which was determined to be sufficiently convergent. To ensure the accuracy and reliability of our results, these calculations were carried out with a lower iterative convergence threshold of 5.0 × $10^{-7}$ eV/atom. In addition, we fully relaxed all atomic coordinates, imposing a limit of 0.02 GPa (safety threshold to prevent the material from reaching its yield strength) on internal stresses and allowing for a maximum displacement of 5.0 × 10${}^{-4}$ Å (the maximum displacement is typically set to maintain the stability and accuracy of the system).

### 2.2. Machine Learning Databases and Models

**Figure 1:**
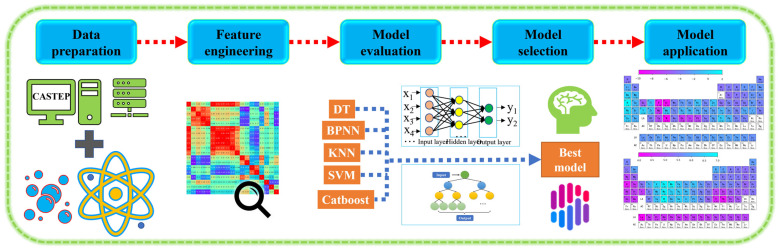
Machine learning steps: feature engineering, machine learning model screening, and result prediction.

The fundamental equation in DFT is the Kohn–Sham equation, which involves solving a set of self-consistent equations for the electron density and Kohn–Sham potential. To enhance the accuracy and efficiency of DFT calculations, machine learning algorithms are employed. These algorithms, such as Back Propagation Neural Network (BPNN) [26], K-Nearest Neighbor (KNN), Support Vector Machine (SVM), Decision Trees (DT), and Catboost, are trained on a dataset of known properties and corresponding electronic structure calculations. By establishing a relationship between input (material descriptors) and output (desired property), these models can accurately and rapidly predict properties, reducing the computational cost associated with DFT calculations. The combination of DFT and machine learning enables the exploration of large materials databases, high-throughput screening, and prediction of the properties of novel materials. By leveraging the computational efficiency of machine learning algorithms and the accuracy of DFT calculations, researchers can accelerate materials discovery and design processes. The flow of machine learning steps is shown in Figure 1.

## 3. Results and Discussion

### 3.1. Database Establishment and Selection of Feature Values

The establishment and selection of feature values within a database are crucial steps in machine learning. The accuracy of machine learning models heavily depends on the quality and relevance of data contained within the database. Therefore, a reliable database is indispensable when developing robust machine learning models. When performing feature selection, it is vital to consider the selection of relevant input variables as they directly affect the predicted output variable, including irrelevant feature values that can lead to overfitting and inaccurate predictions. The crystal structure model, as shown in Figure S1, was computed using first-principles calculations, and the results of this calculation can be found in Table S1. In terms of experimentation, scholars have reported on the comparison of the solution and strengthening effects of alloy elements on the aluminum matrix [4]. According to their research findings, the order of the strengthening effect of alloy elements on aluminum alloy is as follows: Mn > Cu > Si > Zn. These results are in line with our calculations of fracture energy [3], confirming the correctness of the selected unit cell and reliability of the chosen calculation method. In order to gain a better understanding of the data, we conducted a correlation analysis on the DFT calculated data and various descriptors. We calculated the Pearson correlation coefficients between the different features and target values. The formula for the Pearson correlation coefficient is presented below [27,28,29]:

(1) $$\rho_{X,Y}=\frac{\sum \left(X_{i}-\bar{X}\right)\left(Y_{i}-\bar{Y}\right)}{\sqrt{\sum {\left(X_{i}-\bar{X}\right)}^{2}\sum {\left(Y_{i}-\bar{Y}\right)}^{2}}}$$

The Pearson correlation coefficient, denoted as ρ${}_{XY}$, is calculated based on *X*${}_{i}$ and *Y*${}_{i}$, which refer to the eigenvalues and target values, respectively. The values $\bar{X}$ and $\bar{Y}$ represent the average of *X* and *Y*, respectively. The coefficient ranges in magnitude from −1 to 1, where a value of 0 indicates that the two variables are not correlated. The closer the value is to 1 or −1, the stronger the correlation between the data.

**Figure 2:**
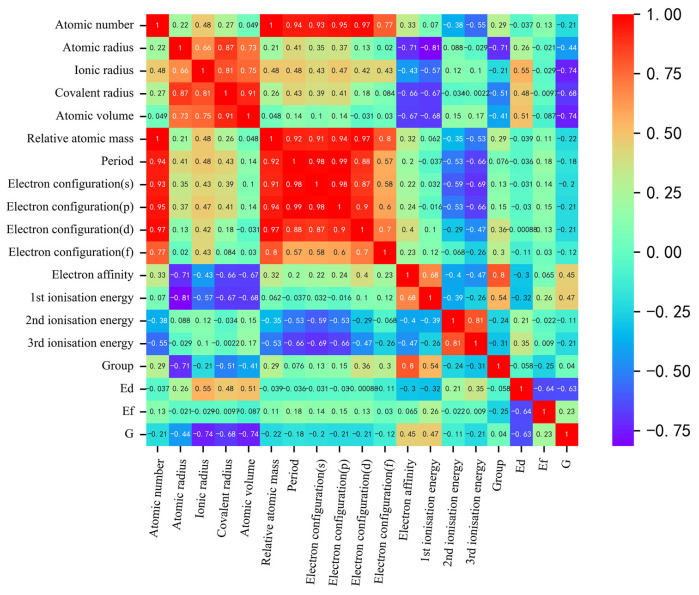
Heat map of the Pearson correlation coefficient matrix between output and input descriptors for the finalized descriptors. The shades of red and blue indicate the strength of positive and negative correlations, respectively.

**Figure 3:**
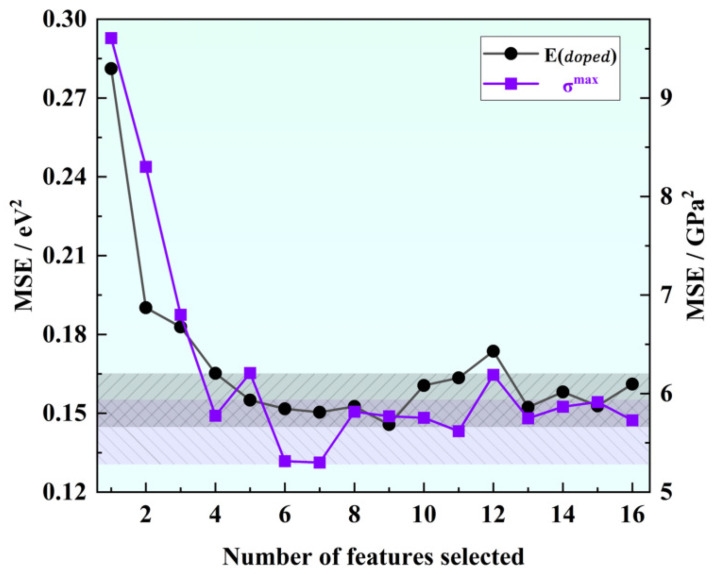
Variation of mean square error with the number of eigenvalues in feature elimination.

As shown in Figure 2, our correlation analysis revealed a strong correlation between certain descriptors, such as atomic number and relative atomic mass, or atomic number and period, and the correlation coefficients exceeded 0.91. Correlation analysis was used for initial feature screening, which allows for the identification of redundant features to avoid overfitting and improving the generalization of the model. In order to mitigate the risk of overfitting by reducing dimensionality, we can appropriately exclude these highly correlated descriptors for simplification purposes. To determine the optimal combination of descriptors and the number of input features for machine learning models predicting solution energy (E${}_{doped}$) and theoretical stress (G), we employed the recursive feature elimination method on the original dataset. This method builds the model iteratively and eliminates features that contribute less to the model’s performance. Using mean square error as the evaluation criterion, we observed that the prediction accuracy for both E${}_{doped}$ and G initially increased and then gradually stabilized as the number of features increased, as shown in Figure 3. The performance, in terms of mean square error, became stable when the number of features reached six. Considering the constraints indicated by the correlation analysis, we ultimately identified the following input features for E${}_{doped}$ (eV): ionic radius, third ionization energy, covalent radius, electron affinity, second ionization energy, first ionization energy, and electron configuration (d). For G (GPa), we identified the following input features: atomic volume, ionic radius, group, second ionization energy, atomic number, first ionization energy, and atomic radius.

**Figure 4:**
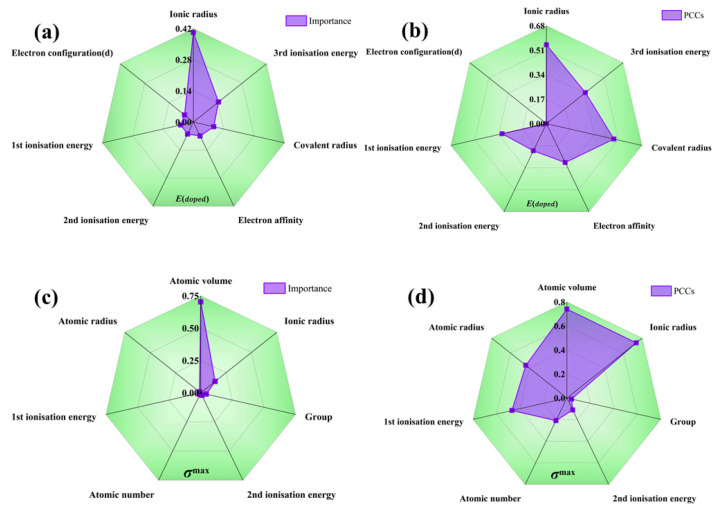
Importance radar chart for (**a**) solution energy and (**c**) theoretical tensile stress, and Pearson correlation coefficients for (**b**) solution energy and (**d**) theoretical tensile stress.

Additionally, the recursive feature elimination method provides a quantitative assessment of the interaction strength among features as shown in Table 1. As seen in Figure 4, the radar plot illustrating feature importance during recursive feature elimination process reveals the following: when predicting E${}_{doped}$, the ionic radius exhibits the most significant influence, with relatively minor disparities in importance among other volume-related features. On the other hand, when predicting G, atomic volume accounts for over 70% importance in relation to the target, and there are substantial differences in importance among the various volume descriptors. It can be seen from Figure 4 that E${}_{doped}$ is relatively high when the radius of the dopant atom differs significantly from the radius of the atom in the crystal. In this case, the position of the dopant atoms in the crystal lattice may result in larger deformations or distortions, which increases the energy. Alloying with small or similar-sized dopant atoms increases the crystal modulus, whereas alloying with large dopant atoms decreases the modulus due to deformations and distortions in the crystal structure, thus affecting the micromechanical behavior of the system. Nonetheless, it must be noted that the recursive feature elimination method solely offers a quantitative measure of importance and does not delve into the specific impact of each descriptor on the target value. This highlights the necessity for further explanations beyond machine learning’s “black box”. Also, the Pearson correlation coefficients (absolute values) of the E${}_{doped}$ and G are presented for reference. Notably, the rankings of importance provided by the correlation coefficients and the recursive feature elimination method do not align perfectly; this discrepancy suggests that these partial descriptions do not adhere to a straightforward linear relationship among the target values. Such findings further demonstrate the imperative need to incorporate machine learning techniques capable of effectively addressing multi-coupling issues.

We initiated a feature screening process to identify potential predictors that could greatly impact the target variables. This was achieved through the calculation of correlation coefficients for each feature against the target variables. Consequently, we observed a weak correlation in the E${}_{doped}$ feature and consequently excluded it from subsequent analysis. In addition, we discovered several strongly correlated features in the dataset. To address concerns regarding multicollinearity, we selected only one feature from each of these groups. Subsequently, we employed a feature elimination method to determine the most relevant input features for predicting E${}_{doped}$ and G. The number of features included was determined based on the evaluation through the root mean square error. Moreover, we utilized importance radar and correlation coefficient plots to visually depict the significance of the relevant features.

Based on the correlation analysis presented above, we employed two sets of data as training inputs for the machine learning model: solution energy with its corresponding descriptor, and theoretical stress with its accompanying descriptor. The descriptor served as the input data for the machine learning dataset, and either solution energy or theoretical stress was treated as the target data. The final selection of feature values to be used as machine learning dataset for E${}_{doped}$ and G used in machine learning were extracted from Tables S2 and S3. In order to ensure consistent scaling of all variables, we normalized both the input and output variables within a range of 0 to 1, using the following mathematical equation [30]:

(2) $$X_{i}^{\prime }=\frac{X_{i}-X_{\min}}{X_{\max}-X_{\min}}$$

where X${}_{i}$ represents the data individual, X${}_{\max}$ is the maximum value in that class of data, and X${}_{\min}$ is the minimum value.

### 3.2. Machine Learning Model Building and Optimization

To accurately assess the performance of various machine learning models when applied to new data and optimize data utilization, cross-validation methods are employed. Cross-validation is a statistical technique that evaluates a model’s ability to generalize by dividing the dataset into distinct partitions. In this section, a commonly used five-fold cross-validation approach was employed. In this approach, the original data is initially randomly divided into eight subsets. Subsequently, the model is trained and validated eight times. During each iteration, the model is trained on seven subsets, constituting the training set, and then tested on the remaining subset, the validation set. This process is repeated eight times, and the results are averaged to obtain more precise estimates of the model’s performance. We implemented the aforementioned algorithms in Python, utilizing scientific computing packages such as pandas and numpy. In order to assess the effectiveness of various models, we introduced mean square error (MSE), mean absolute percentage error (MAPE), and coefficient of determination (R${}^{2}$) as evaluation metrics. The calculations for these metrics are outlined as follows [31]:

(3) $$\mathrm{MSE}=\frac{1}{N}\sum_{i=1}^{N}{\left(y_{i}-\hat{y}\right)}^{2}$$

(4) $$\mathrm{RMSE}=\sqrt{\frac{1}{N}\sum_{i=1}^{N}{\left(y_{i}-\hat{y}\right)}^{2}}$$

(5) $$\mathrm{MAPE}=\frac{1}{N}\sum_{i=1}^{N}\left|\frac{y_{i}-{\hat{y}}_{i}}{y_{i}}\right|\times 100\%$$

(6) $$R^{2}=1-\frac{\sum_{i=1}^{N}{\left(y_{i}-\hat{y}\right)}^{2}}{\sum_{i=1}^{N}{\left(y_{i}-\bar{y}\right)}^{2}}$$

where $\hat{y}$${}_{i}$ is the ML algorithm predicted value, *y*${}_{i}$ is the DFT calculated value, $\bar{y_{i}}$ is the mean of the DFT calculated value, and *N* is the number of samples.

**Figure 5:**
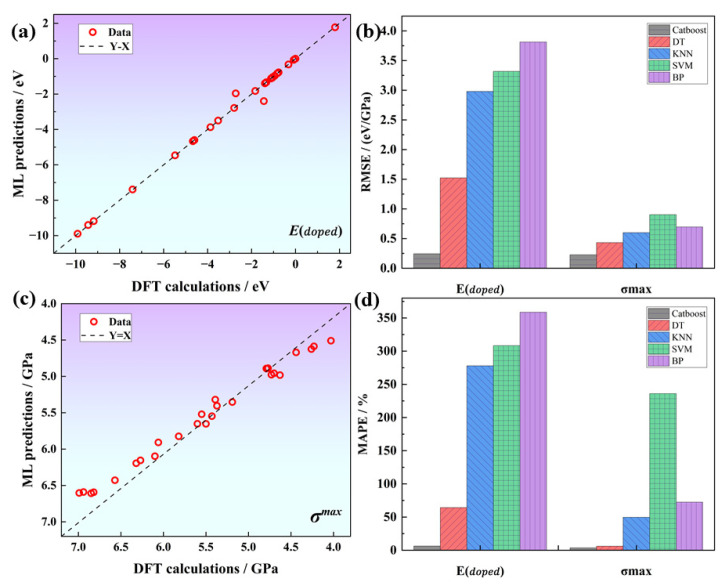
Catboost regression plots for (**a**) E${}_{doped}$ and (**c**) G; (**b**,**d**) show the comparison of RMSE and MAPE for differents models.

The cross-validation results of different algorithms indicate that the tree algorithm significantly outperforms the other algorithms, whereas the BPNN algorithm performs the worst, as shown in Table 2. These results could be attributed to the fact that the neural network requires a large number of training parameters and is not suitable for this small sample data problem. Within the category of tree-based algorithms, Catboost demonstrates a significant advantage over the traditional DT algorithm. It achieves a RMSE (root mean square error) of 0.24 and 0.22, as well as coefficients of determination of 0.99 and 0.93 for the E${}_{doped}$ and G predictions, respectively. Regression analysis confirms that the machine learning predicted values are significantly correlated with the DFT calculated values, and the data points are evenly distributed around the identity line (Y = X), suggesting that our model fits the data well, as shown in Figure 5. The prediction errors for E${}_{doped}$ and G are 3.64% and 3.63%, respectively, which meet the target accuracy requirements. Therefore, we select the Catboost algorithm as the model for subsequent machine learning tasks.

After conducting a comprehensive cross-validation evaluation, we determined that the Catboost model exhibited significantly superior performance. Consequently, we decided to employ Catboost as our final prediction model. In order to explore the most effective algorithms for small sample data problems, we experimented with a variety of techniques, including tree algorithms and neural network algorithms. Specifically, we evaluated the performance of Catboost, Decision Trees, Back Propagation Neural Network, K-Nearest Neighbor, and Support Vector Machine. Tree algorithms utilize a tree structure to make data-based decisions and predictions. When addressing small sample problems, tree algorithms offer several advantages, including simplicity, ease of implementation, and robustness. As a result, they are commonly regarded as an appealing choice in such scenarios.

### 3.3. Interpretable Machine Learning and Result Prediction

**Figure 6:**
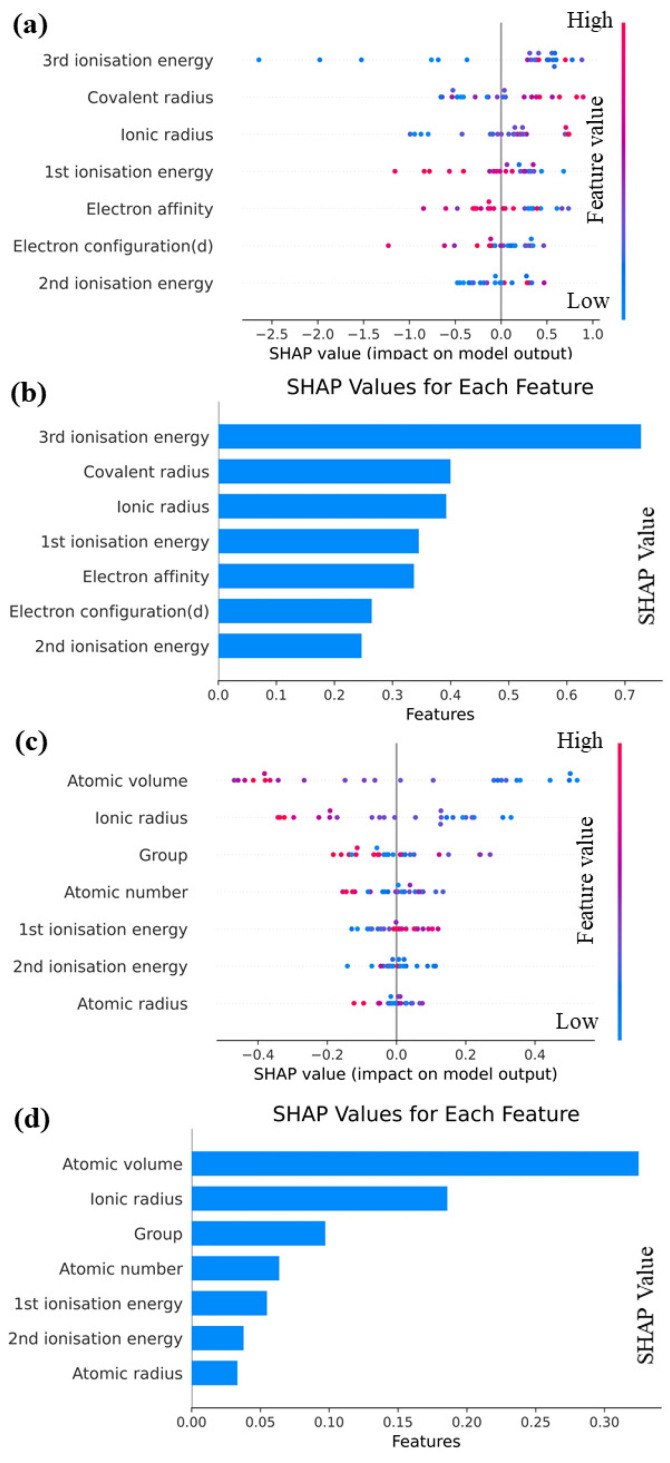
(**a**) SHAP overall feature map and (**b**) SHAP feature importance map for E${}_{doped}$; (**c**) SHAP overall feature map and (**d**) SHAP feature importance map for G.

The importance of interpretable machine learning is discussed in this section, and Shapley additive explanations (SHAP) [32] are employed to analyze the Catboost model. The goal is to achieve a comprehensive understanding of the impact of each feature on prediction outcomes. A structured and systematic approach that employs machine learning techniques is introduced in this study to predict performance parameters for various elements. By conducting feature engineering, model selection, and interpretability, a highly accurate prediction model is constructed, providing deep insights into its functioning. Valuable contributions to materials science are provided by this research and enhances our understanding of interactions among different elements. To gain a comprehensive understanding of the functionality of our predictive model, it is crucial to employ SHAP as a tool to explain complex machine learning models. Using this approach, the influence of each feature on the model’s prediction outcome is comprehended, thus improving transparency and reliability. The facilitation of new scientific discoveries is particularly valuable in complex, multi-coupled systems due to such transparency. Therefore, the SHAP approach is utilized to elucidate the Catboost model. As illustrated by the feature map shown in Figure 6, each row represents a feature, and each point represents a sample. The magnitude of the feature value is indicated by the color intensity, ranging from red to blue, with larger values represented by redder colors and smaller values denoted by bluer colors. The feature importance graph provides a ranking of feature importance based on the average absolute SHAP value for each feature.

It can be see from Figure 6a,b that the overall SHAP eigenplot of E${}_{doped}$ reveals that almost all eigenvalues exhibit a linear relationship with E${}_{doped}$ to some extent. Specifically, an increase in the covalent radius, ionic radius, and second ionization energy corresponds to an increase in E${}_{doped}$, whereas an increase in first ionization energy, electron configuration (d), and electron affinity leads to a decrease in E${}_{doped}$. It can be seen from Figure 6 that third ionization energy exerts the greatest effect on E${}_{doped}$, whereas the importance of other features does not significantly vary. Moreover, the SHAP values of nearly all the feature quantities exceed 0.25, indicating their substantial impact on E${}_{doped}$. From Figure 6c,d, we can see that the overall SHAP feature plot of G demonstrates that an increase in atomic volume and ionic radius leads to an increase in G, whereas an increase in first ionization energy results in a decrease in G. A more complex non-linear relationship is observed between atomic radius and group and G, with blue data points distributed at both ends and red data points concentrated in the middle. This distribution indicates that extremely large or small eigenvalues reduce the valuation of G, and the negative impact of excessively large eigenvalues is more pronounced. The SHAP feature importance plot of G reveals that different features exert significantly different effects on the degree of existence of G. Notably, the SHAP values of atomic volume and ionic radius are significantly larger than those of various other descriptors. Among these descriptors, atomic volume emerges as the most crucial predictor for G, whereas atomic radius is the least significant.

**Figure 7:**
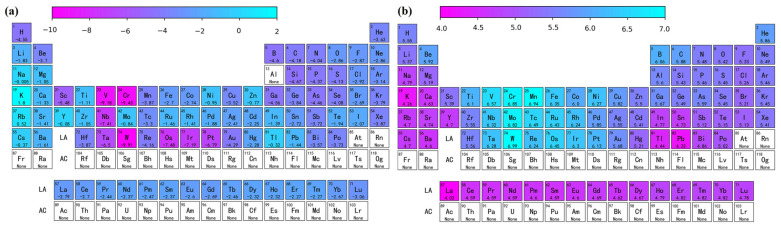
Predicted diagram of the periodic table of elements showing (**a**) solution energy and (**b**) theoretical tensile stress.

The solution energy and theoretical stress of the remaining other atoms in the aluminum matrix for the full periodic table data were predicted using the Catboost model, and a heat map was plotted, as shown in Figure 7. A structured and systematic approach is provided in this paper for predicting the performance parameters of different elements using machine learning. As shown in Figure 7a, the elements with relatively low Edoped were Sc, W, Ta, etc. Figure 7b shows that the elements with significant enhancement of the aluminum matrix in terms of theoretical stresses were Sc, Ni, W, Mn, etc. By performing feature engineering, model selection, and interpretable work, not only did we develop a highly accurate prediction model, but we also gained an in-depth understanding of how to achieve these results. Using a machine learning accelerated first-principles approach provides a theoretical basis for further design of novel aluminum alloys.

## 4. Conclusions

In this paper, we conducted a comprehensive study on the effects of alloying atoms on the stability and micromechanical properties of aluminum alloys using a machine learning accelerated first-principles approach. The preliminary work involved exploring high-throughput first-principles calculations and extracting fundamental data, such as solution energy and theoretical stress, for atomically doped aluminum substrates. The machine learning dataset was constructed using data from previous high-throughput computational work, incorporating features such as atomic radius, ionic radius, and first ionization energy. Feature elimination was implemented to enhance model accuracy and efficiency. We compared the performance of five different algorithms, ultimately selecting the Catboost model based on its lowest RMSE of 0.24 and lowest MAPE of 6.34. Through this comparison of different machine learning algorithms, the Catboost model emerged as the superior choice and was utilized as the final prediction model. Additionally, the SHAP was employed for interpretative analysis, enabling a deeper understanding of how each feature contributes to the prediction results. Furthermore, our approach facilitated the prediction of alloying stability and micromechanical behavior for various elements in the full periodic table on an aluminum matrix. The results showed that alloying atoms such as K, Na, Y, and Tl were difficult to solid-solve into the aluminum matrix. However, alloy atoms such as Sc, Cu, B, Zr, Ni, Ti, Nb, V, Cr, Mn, Mo, and W were found to contribute to the strengthening of aluminum alloys. Theoretical investigations into solid solutions and the strengthening effects of various alloying atoms in an aluminum matrix provide valuable insights for selecting suitable alloy elements. In conclusion, our work presents an interpretable machine learning accelerated first-principles research methodology that provides a theoretical basis for the development of new aluminum alloys.

## Figures and Tables

**Table 1:** The value of the change in mean square error of E${}_{doped}$ and G with the change in eigenvalues in the feature elimination method.

N	1	2	3	4	5	6	7	8	9	10	11	12	13	14	15	16
E${}_{doped}$ (eV${}^{2}$)	0.28	0.19	0.18	0.16	0.16	0.15	0.15	0.15	0.14	0.16	0.16	0.17	0.15	0.15	0.15	0.16
G (GPa${}^{2}$)	9.61	8.30	6.80	5.78	6.20	5.31	5.30	5.82	5.76	5.75	5.62	6.19	5.74	5.86	5.91	5.73

**Table 2:** Mean square error, root mean square error, and MAPE corresponding to different machine learning models for E${}_{doped}$ and G.

			E${}_{\mathrm{doped}}$					G		
	Catboost	DT	BPNN	KNN	SVM	Catboost	DT	BPNN	KNN	SVM
MSE	0.06	2.32	14.53	8.87	11.00	0.05	0.18	0.48	0.36	0.81
RMSE	0.24	1.52	3.81	2.98	3.31	0.22	0.43	0.69	0.59	0.90
MAPE (%)	6.34	64.2	358.6	277.93	308.39	3.63	6.11	72.38	49.61	236.03

## Data Availability

Data is contained within the article or Supplementary Material.

## Supplementary Materials

The following supporting information can be downloaded at: https://www.mdpi.com/article/10.3390/ma16206757/s1. Figure S1: Schematic diagram of the crystal structure calculated by first principles, where the green spheres are dopant atoms and the purple spheres are aluminum atoms. Figure S2: Comparison of formation energies of alloy atom doped aluminum matrix for machine learning prediction results with first principle calculations. Figure S3: Comparison of theoretical tensile stress for machine learning prediction results with first principle calculations. Figure S4: Test for convergence of total energy with (a) cutoff energy and (b) k-points grid mesh. Figure S5: Crystal structure of (a) perfect aluminum and (b) X-doped aluminum. Table S1: Solution energy (Edoped) as well as theoretical stresses (G) and lattice constants (a,b,c) for the alloy atom doped aluminum matrix system. Table S2: Final selection of feature values to be used as machine learning dataset for Ed. Table S3: Final selection of feature values to be used as machine learning dataset for G.
