# Supplementary material for: Exploration of Solid Solutions and the Strengthening of Aluminum Substrates by Alloying Atoms: Machine Learning Accelerated Density Functional Theory Calculations
Source: Materials (Basel). 2023 Oct 19;16(20):6757. doi: 10.3390/ma16206757 (PMC10608410; doi:10.3390/ma16206757)
Supplement: Supplementary file 1 [file materials-16-06757-s001.zip › materials-2642358-supplementary.pdf]

Supplemental Information

Exploration of solid solution and strengthening of aluminum substrates by alloying atoms: Explainable machine learning accelerated density generalized theory calculations

Jingtao Huang [1,†], Jingteng Xue [1,†], Mingwei Li [2], Yuan Cheng [3], Zhonghong Lai [4], Jin Hu [1], Fei Zhou [5], Nan Qu [1], Yong Liu [1,2,*] and Jingchuan Zhu [1,*]

[1] School of Materials Science and Engineering, Harbin Institute of Technology, Harbin 150001, China;

[2] National Key Laboratory for Precision Hot Processing of Metals, Harbin Institute of Technology, Harbin 150001, China

[3] National Key Laboratory of Science and Technology on Advanced Composites in Special Environments, Harbin Institute of Technology, Harbin 150001, China

[4] Center for Analysis, Measurement and Computing, Harbin Institute of Technology, Harbin 150001, China;

[5] State Key Laboratory for Environment-Friendly Energy Materials, School of Materials Science and Engineering, Southwest University of Science and Technology, Mianyang 621010, China

***** Correspondence: lyonghit@hit.edu.cn (Y.L.); fgms@hit.edu.cn (J.Z.)

[†] These authors contributed equally to this work.

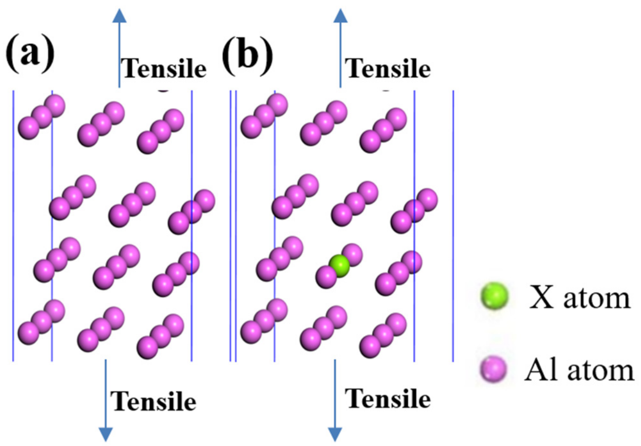

Figure S1 Schematic diagram of the crystal structure calculated by first principles, where the green spheres are dopant atoms and the purple spheres are aluminum atoms.

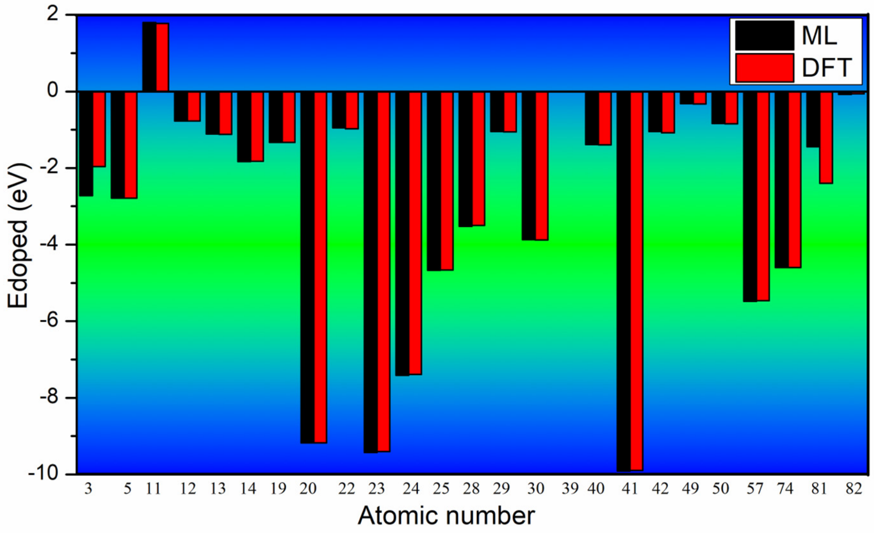

Figure S2 Comparison of formation energies of alloy atom doped aluminum matrix for machine learning prediction results with first principle calculations.

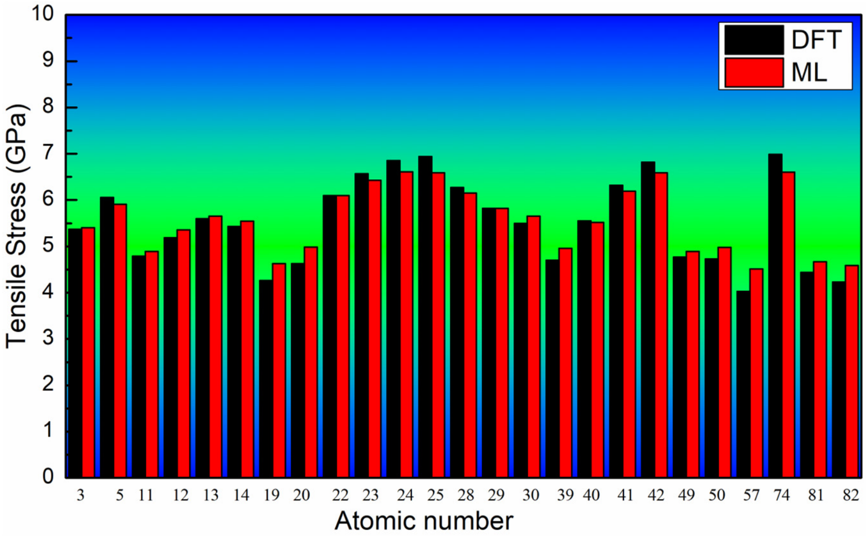

Figure S3 Comparison of theoretical tensile stress for machine learning prediction results with first principle calculations.

Table S1 Solution energy (E_{doped}) as well as theoretical stresses (G) and lattice constants (a,b,c) for the alloy atom doped aluminum matrix system.

N_a	Atomic symbols	E_{doped}(eV)	G(GPa)	a(Å)	b(Å)	c(Å)
3	Li	-2.72	5.37	8.43	8.43	37.06
5	B	-2.79	6.06	8.42	8.42	37.11
11	Na	1.8	4.79	8.44	8.44	37.27
12	Mg	-0.77	5.19	8.44	8.44	37.17
13	Al	-1.11	5.6	8.44	8.44	37.02
14	Si	-1.83	5.43	8.43	8.43	37.05
19	K	-1.33	4.26	8.47	8.47	37.52
20	Ca	-9.18	4.63	8.46	8.46	37.39
22	Ti	-0.95	6.1	8.45	8.45	36.97
23	V	-9.43	6.57	8.44	8.44	36.83
24	Cr	-7.41	6.85	8.43	8.43	36.74
25	Mn	-4.67	6.94	8.43	8.43	36.72
28	Ni	-3.52	6.27	8.41	8.41	36.92
29	Cu	-1.05	5.82	8.42	8.42	37.00
30	Zn	-3.87	5.5	8.43	8.43	37.04
39	Y	-0.005	4.7	8.47	8.47	37.40
40	Zr	-1.39	5.55	8.46	8.46	37.14
41	Nb	-9.91	6.32	8.45	8.45	36.94
42	Mo	-1.05	6.82	8.44	8.44	36.83
49	In	-0.32	4.77	8.46	8.46	37.32
50	Sn	-0.84	4.73	8.46	8.46	37.37
57	La	-5.48	4.03	8.49	8.49	37.57
74	W	-4.6	6.99	8.44	8.44	36.83
81	Tl	-1.44	4.44	8.46	8.46	37.43
82	Pb	-0.08	4.23	8.47	8.47	37.51

Table S2 Final selection of feature values to be used as machine learning dataset for Ed

N_a	Ionic radius(Å)	Covalent radius	Electron configuration(d)	Electron affinity	1st ionisation energy	2nd ionisation energy	3rd ionisation energy
3	0.69	1.41	20	1.96	708.6	1411.8	2943
5	1.061	1.69	21	1.1	538.1	1067	1850.3
11	1.38	2.03	0	0.82	418.8	3052	4420
12	0.74	1.25	10	1.65	906.4	1733.3	3833
13	0.605	1.32	2	1.54	658.8	1309.8	2652.5
14	0.76	1.23	0	0.98	520.2	7298.1	11815
19	0.99	1.74	0	1	589.8	1145.4	4912.4
20	0.59	1.22	3	1.63	650.9	1414	2830
22	0.69	1.15	8	1.91	737.1	1753	3395
23	0.52	1.18	5	1.66	652.9	1590.6	2987
24	0.69	1.34	14	1.6	652.1	1380	2416
25	0.4	1.11	0	1.9	786.5	1577.1	3231.6
28	0.73	1.17	10	1.9	745.5	1957.9	3555
29	0.72	1.36	0	1.31	737.7	1450.7	7732.7
30	0.46	1.17	5	1.55	717.3	1509	3248
39	1.02	1.54	0	0.93	495.8	4562	6910.3
40	0.8	1.44	20	1.78	558.3	1820.7	2704
41	0.62	1.3	24	1.7	770	1700	0
42	0.72	1.45	12	1.33	640.1	1270	2218
49	1.5	1.48	30	1.8	589.4	1971	2878
50	0.65	1.3	15	2.16	684.3	1560	2618
57	0.745	1.44	1	1.36	633.1	1235	2388.6
74	0.23	0.82	0	1.92	800.6	2427.1	3659.7
81	1.19	1.47	30	1.8	715.6	1450.5	3081.5
82	0.9	1.62	11	1.22	600	1180	1980

Table S3 Final selection of feature values to be used as machine learning dataset for G

N_a	Atomic number	Atomic radius	Ionic radius	Atomic volume	1st ionisation energy	2nd ionisation energy	Group
3	3	2.05	0.76	13.1	520.2	7298.1	1
5	5	1.17	0.23	4.6	800.6	2427.1	13
11	11	2.23	1.02	23.7	495.8	4562	1
12	12	1.72	0.72	13.97	737.7	1450.7	2
13	13	1.82	0.535	10	577.5	1816.7	13
14	14	1.46	0.4	12.1	786.5	1577.1	14
19	19	2.77	1.38	45.46	418.8	3052	1
20	20	2.23	0.99	29.9	589.8	1145.4	2
22	2	0.605	10.64	658.8	1309.8	4	6.1
23	1.92	0.59	8.78	650.9	1414	5	6.57
24	1.85	0.52	7.23	652.9	1590.6	6	6.85
25	1.79	0.46	1.39	717.3	1509	7	6.94
28	1.62	0.69	6.59	737.1	1753	10	6.27
29	1.57	0.73	7.1	745.5	1957.9	11	5.82
30	1.53	0.74	9.2	906.4	1733.3	12	5.5
39	2.27	0.9	19.8	600	1180	3	4.7
40	2.16	0.72	14.1	640.1	1270	4	5.55
41	2.08	0.69	10.87	652.1	1380	5	6.32
42	2.01	0.65	9.4	684.3	1560	6	6.82
49	2	0.8	15.7	558.3	1820.7	13	4.77
50	1.72	0.69	16.3	708.6	1411.8	14	4.73
57	2.74	1.061	20.73	538.1	1067	0	4.03
74	2.02	0.62	9.53	770	1700	6	6.99
81	2.08	1.5	17.2	589.4	1971	13	4.44
82	1.81	1.19	18.17	715.6	1450.5	14	4.23

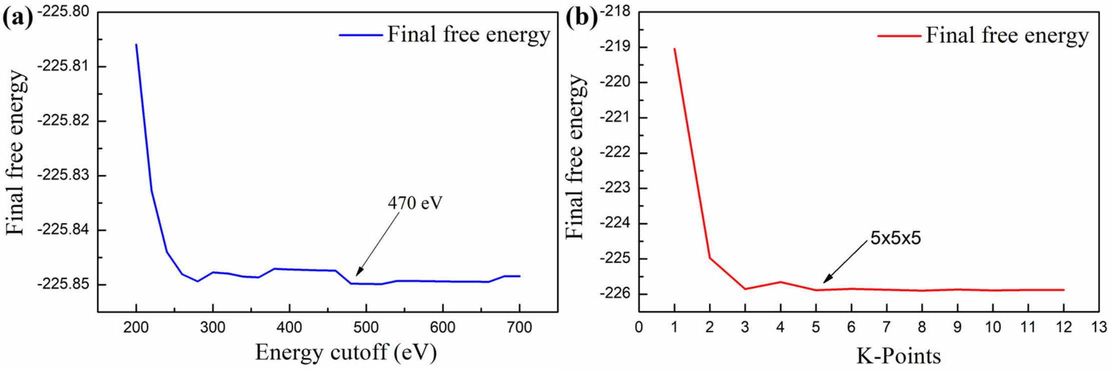

Figure S4 Test for convergence of total energy with (a) cutoff energy and (b) k-points grid mesh.

Convergence test:
When the energy as a function of the lattice strain is used to gain the elastic constants of a single crystal, the calculations require a very high degree of precision because the energy differences involved are of the order of several meV. This circumstance requires the use of a fine k-point mesh and a large energy cutoff. Therefore, the convergence of the total energy with respect to both the k-point sampling and the energy cutoff have been tested as shown in Fig. S4(a) and Fig. S4(b), respectively. With larger energy cutoff or more k points, the changes of total energy of the system were less than 0.001 eV. As a result, the electronic wave functions were expanded using a plane-wave basis set with cutoff energy of 470 eV. The Brillouin-zone sampling was performed with a 5×5×5 Mokhorst-Pack k-point grid mesh.

Solution Energy:

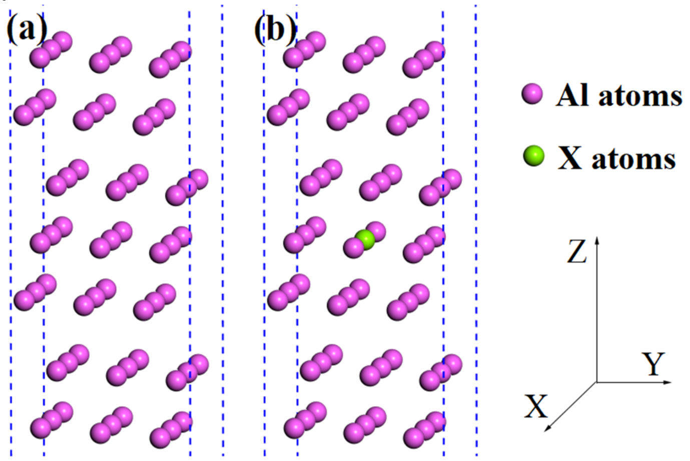

Figure S5 Crystal structure of (a)perfect aluminum and (b)X-doped aluminum.
The alloying of alloying atoms is calculated using the Al72X1 model, as shown in Fig. S5. The unit cell consists of 71 Al atoms and 1 dopant atom X. The Al atoms are represented by pink, while the dopant atoms X are represented by cyan balls. The alloying ratio of the model is 1.38 at.%. To compare the stability of doped systems with various alloying elements, the formation energies of different alloying element-doped aluminum substrates are calculated using the following solution energy formula. The formation energy formula is expressed as follows:

$$E_{doped} = E_{all} - E_{pure} + \mu_{Al} - \mu_{doped} \quad (S1)$$

Among them, E_{all} is the total energy of the alloy element alloying system. E_{pure} is the total energy of a perfect aluminum crystal before alloying. μ_{Al} is the energy of one aluminum atom, μ_{doped} is the energy of doped alloy atoms.

Theoretical stress:

The interface strength plays a significant role in determining the mechanical strength and plasticity of alloys, and it is an important parameter in engineering applications. The distances are set at 0.05, 0.10, 0.15, and 0.90 nm. In a fully relaxed system, each relaxation layer is uniformly stretched with

a step size of 0.005 nm. When the interface is about to fracture, each relaxation layer is uniformly stretched with a step size of 0.001 nm. The fracture energy and maximum tensile stress [20] can be obtained using the following equation [30]:

$$E_{frac} = \frac{E_{sg} - E_{total}}{S} \qquad \text{(S2)}$$

in this equation, E_{sg} represents the total energy of the system at the point of tensile fracture, while E_{total} represents the total energy of the system without any tensile deformation.

The maximum stress of the system is determined by the function f(x), which is a fitting of fracture energy with separation distance. In the first principles calculations that we employ, we denote the input as x in the formulas for fracture energy and theoretical stress. This input corresponds to the distance between separations observed during simulated stretching. By considering this distance, the formulas can accurately quantify the fracture energy and theoretical stress in our calculations.

$$\mathrm{f(x)} = E_{frac} - E_{frac}(1 + \frac{x}{\lambda})e^{-x/\lambda} \qquad \text{(S3)}$$

In this equation, λ represents the separation distance. This function, proposed by Rose *et al.* [31], is known as the universal binding curve and is used to describe the bonding characteristics between atoms. It establishes the best fit relationship between metal bonding energy and atomic distance. By taking the derivative of f(x), the tensile stress can be expressed as follows:

$$f'(x) = \frac{E_{frac}x}{\lambda^2} e^{-x/\lambda} \qquad \text{(S4)}$$

When x is equal to λ, we can obtain the theoretical maximum tensile stress (σ^{max}):

$$\sigma^{max} = f'(x) = \frac{E_{frac}x}{\lambda^2} e^{-x/\lambda} = \frac{E_{frac}}{\lambda e} \qquad \text{(S5)}$$

Feature Elimination:

In each iteration, the least important feature is removed from the feature set. After removing the feature, the feature evaluation is performed again, reordered and the least important feature is removed. This process is repeated until the number of iterations is reached. The error in this process will change with the change in the number of features. By comparing the model accuracy and referring to the results of correlation analysis for screening, we determine the best input features for different target values. However, since correlation analysis only considers linear correlation, it is only used to remove highly correlated input features.
